# Diagnostic Value of Sural Nerve Biopsy: Retrospective Analysis of Clinical Cases From 1981 to 2017

**DOI:** 10.3389/fneur.2019.01218

**Published:** 2019-11-22

**Authors:** Valeria Prada, Sara Massucco, Consuelo Venturi, Alessandro Geroldi, Emilia Bellone, Paola Mandich, Michele Minuto, Emanuela Varaldo, Giovanni Mancardi, Marina Grandis, Angelo Schenone

**Affiliations:** ^1^Department of Neurosciences, Rehabilitation, Ophthalmology, Genetics and Maternal/Child Sciences, University of Genoa, Genoa, Italy; ^2^Department of Neurology, Policlinico San Martino IRCCS, Genoa, Italy; ^3^Department of Surgical Sciences (DISC), University of Genoa, Genoa, Italy; ^4^Department of Surgery, Policlinico San Martino IRCSS, Genoa, Italy; ^5^Fondazione Maugeri ICS, Genova, Italy

**Keywords:** sural nerve biopsy, vasculitic neuropathy, amyloidotic neuropathy, neuropathy, axonal neuropathies, demyelinating neuropathies

## Abstract

Nerve biopsy represents the conclusive step in the diagnostic work-up of peripheral neuropathies, and its diagnostic yield is still debated. The aim of this study is to consider the impact of nerve biopsy on reaching a useful diagnosis in different peripheral neuropathies and its changing over time. We retrospectively analyzed 1,179 sural nerve biopsies performed in the period 1981–2017 at Neurological Clinic of Policlinico San Martino (Genoa). We relied on medical records and collected both clinical and pathological data in a database. Biopsy provided univocal diagnoses in 53% of cases (with an increase over time), multiple diagnostic options in 14%, while diagnosis was undetermined in 33% (undetermined reports decreased during the years). In 57% of patients, the pre-biopsy suspicion was confirmed, while in 43% sural biopsy modified the clinical diagnosis. The highest yield was in axonal neuropathies (29% undetermined reports vs. 40% in demyelinating and 48% in mixed neuropathies). In 68% of patients with vasculitic neuropathy, this etiology was already suspected, whereas in 32% nerve biopsy modified the clinical diagnosis. During the years, the number of annually performed biopsies decreased significantly (*p* = 0.007), with an increase in the mean age of patients (*p* < 0.0001). The percentage of hereditary neuropathies had a significant decrease (*p* = 0.016), while the rate of vasculitic and chronic inflammatory neuropathies increased (*p* < 0.0001). This is the largest Italian study addressing the yield of sural nerve biopsy. During the years, we observed a progressive refinement of the indication of this procedure, which confirms its utility for interstitial neuropathies, particularly if non-systemic vasculitic neuropathy is suspected.

## Introduction

Peripheral neuropathies represent one of the main neurological diseases, with a prevalence of 2.4% in general population, reaching 8% in people older than 55 (1). The diseases that can lead to a polyneuropathy are more than one hundred (2). Diagnosis is usually achieved by means of medical history, physical examination, electrophysiology, laboratory tests, and possibly cerebrospinal fluid examination, imaging, and genetic testing (1, 3–5). Sural nerve biopsy usually represents the conclusive step in the diagnostic work-up of several peripheral neuropathies. It is an invasive procedure, so it is applied only in cases unresolved after an extensive workout; when successful it can modify the subsequent therapeutic choices (5–7). The main consequence of biopsy is an area of cutaneous anesthesia at the lateral margin of foot, in the territory previously innervated by sural nerve. Major complications, such as neuroma formation or wound infections occur in 1% of patients, moreover in patients affected by vasculitis receiving corticosteroid therapy, this may result in delayed healing (8–12). It is known and demonstrated that only few peripheral neuropathies require biopsy (7), but in some cases this procedure may be indispensable for diagnosis (5). Currently, the main indication of nerve biopsy is restricted to the investigation of treatable causes of neuropathy. In effect, this procedure is particularly useful to diagnose interstitial neuropathies, such as vasculitis, granulomatosis, leprosy, amyloidosis or tumors, but also to confirm a chronic inflammatory demyelinating polyneuropathy with atypical presentation (6, 7).

Clinical utility (5, 13, 14), indications, timing, site (15–20), and execution methods of nerve biopsy are still a topic of discussion within clinical and scientific community (6, 7, 21–24). While some authors emphasize the importance of biopsy in cryptogenic neuropathies (12), others do not. According to most authors, the main indication for nerve biopsy is the suspicion of vasculitic neuropathy (7, 12). Identifying groups of patients that should not undergo this procedure would lower the percentage of uninformative biopsies (12). The extensive series of cases at the Neurological Clinic of Policlinico San Martino (Genoa) is a unique asset to answer these questions. In fact, over a period of 37 years, more than a thousand biopsy samples were examined. Moreover, since 2018, the Neuropathology Laboratory has become a real Biobank, devoted to both maintenance and sharing of biological material for diagnostic and research purposes. The first question we wanted to answer with this study is the diagnostic return of nerve biopsy in our neuropathology laboratory. The second question was to evaluate how the behavior of clinicians and neuropathologists dedicated to interpretation of histological material has changed over the years. We also decided to evaluate how indications of nerve biopsy have changed during the years, in order to highlight the cases in which biopsy can still have a diagnostic meaning. Finally, we analyzed the correspondence between the histological and clinical suspicion in order to evaluate the impact of nerve biopsy in modifying the pre-biopsy diagnosis.

## Materials and Methods

**Figure 1:**
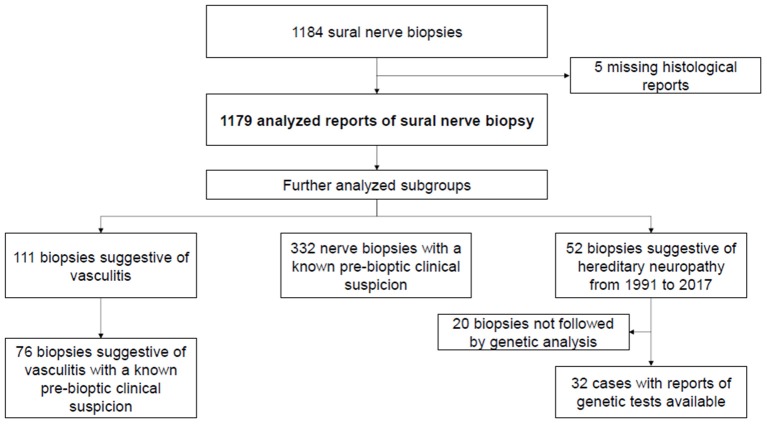
Selection criteria.

We retrospectively analyzed 1,184 medical records of the sural nerve biopsies performed in the period 1981–2017 at the Neuropathology Laboratory of Neurological Clinic of Policlinico San Martino (Genoa). Excluding 5 missing histological reports, we analyzed 1,179 reports of sural nerve biopsy and collected both clinical and pathological data in a database Excel format (data are summarized in Figure 1).

In the presence of a precise pre-biopsy clinical suspicion, we calculated the percentage of patients in whom this suspicion was confirmed by histological examination and in which instead the biopsy modified the clinical diagnosis. Considering the cases in which histology did not provide diagnostic indications, we assessed what the clinical suspicion was and we focused on the yield of biopsy in neuropathies of unknown origin.

Regarding patients with a histological picture of vasculitis, we analyzed the available medical records to assess what was the initial hypothesis and the percentage of different types of vasculitis encountered.

Considering the patients with histologically diagnosed hereditary neuropathy since 1991, year of identification of 17p11.2 chromosome duplication as responsible for Charcot Marie Tooth type 1A (CMT1A) (25, 26), we assessed the subsequent molecular confirmation of the diagnosis. We then looked for these patients on a database containing the genetic test reports.

We used linear regression and Spearman correlation coefficient R to analyze the decrease of the number of annually performed biopsies, the increase in the average age of patients and the reduction of the percentage of minors. We also evaluated the trend of the percentage of indeterminate reports and univocal diagnoses over the years. Finally, we considered whether certain categories of neuropathy, in particular hereditary, toxic-deficient, chronic inflammatory and vasculitic forms, showed either a decrease or an increase. Results were considered statistically significant in the presence of *p* < 0.05.

## Results

**Figure 2:**
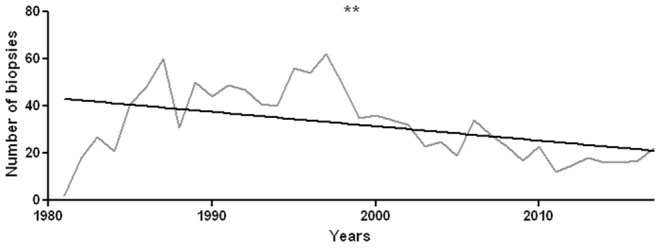
Number of sural nerve biopsies performed during each year from 1981 to 2017. There is a statistically significant decrease in the number of annually performed histological investigations, with *r*^2^ = 0.19 and *p* = 0.007. **p < 0.01.

The total number of analyzed biopsies is 1,179. The number of annually performed biopsies has undergone a significant decrease (*r*^2^ = 0.19, *p* = 0.007, Figure 2).

**Figure 3:**
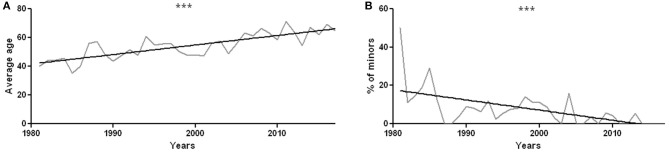
**(A)** Average age of patients undergoing sural nerve biopsy during each year from 1981 to 2017. The increase in mean age is statistically significant with *r*^2^ = 0.64 and *p* < 0.0001. **(B)** Percentage of minors compared to all the patients undergoing sural nerve biopsy year by year. The decrease in the percentage of biopsies performed on minors is statistically significant, with *r*^2^ = 0.35 and *p* = 0.0001. ***p < 0.001.

The mean age of patients is 46 years (range 5 months to 87 years, Supplementary Material for a data resume). The average age of patients who underwent sural nerve biopsy has significantly increased over the years (*r*^2^ = 0.64, *p* < 0.0001, Figure 3A). There were 87 minors (0–17 years old), including 57 males and 30 females. The percentage of minors significantly decreased from 1981 to 2017 (*r*^2^ = 0.35, *p* = 0.0001, Figure 3B).

The univocal diagnoses were 52.7% (*n* = 621), the cases in which biopsy provided multiple diagnostic options were 13.7% (*n* = 162), and indeterminate reports were 33.6% (*n* = 396).

**Figure 4:**
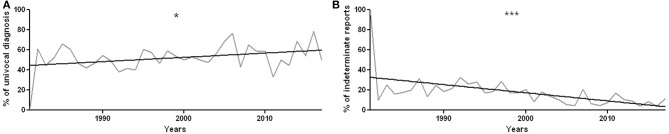
**(A)** Percentage of definite diagnoses year by year, from 1981 to 2017. There is a statistically significant increase in univocal diagnoses, with *r*^2^ = 0.12 and *p* = 0.04. **(B)** Percentage of undetermined reports over the years. The decrease in the percentage of undetermined reports is statistically significant, with *r*^2^ = 0.3010 and *p* = 0.0004. *p < 0.05 and ***p < 0.001.

During the years, the percentage of unique diagnoses increased (*r*^2^ = 0.12, *p* = 0.04, Figure 4A), while the percentage of indeterminate histological findings significantly decreased (*r*^2^ = 0.3, *p* = 0.0004, Figure 4B).

In axonal neuropathies, some diagnostic indication was obtained in 71% of cases (*n* = 281), while the report was indeterminate in the remaining 29% (*n* = 114). Regarding demyelinating neuropathies, instead, histological examination provided diagnostic indications in 60.5% (*n* = 245) and did not contribute to the achievement of an etiological diagnosis in 39.5% of cases (*n* = 160). In mixed neuropathies, in which it was therefore not possible to identify whether the pathological process was initially axonal or demyelinating, the reports were indeterminate in 48% of cases (*n* = 106), while it was possible to formulate diagnostic hypotheses in the remaining 52% (*n* = 115).

Considering the 87 minors, nerve was normal in 18 cases, histological report was indeterminate in 32, genetic neuropathy was diagnosed in 30, and dysimmune neuropathy in 2 patients (1 Guillain-Barré syndrome and 1 chronic relapsing idiopathic polyneuritis). In the remaining 5 cases a double diagnostic hypothesis was formulated, i.e., hereditary neuropathy or chronic inflammatory demyelinating polineuropathy (CIDP).

**Figure 5:**
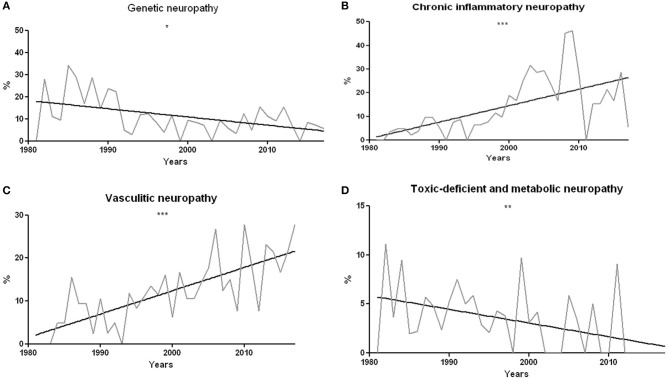
**(A)** Percentage of genetic neuropathies compared to the total of univocal diagnostic indications over the years. The decrease is statistically significant, with *r*^2^ = 0.26 and *p* = 0.016. **(B)** Percentage of chronic inflammatory neuropathies compared to the total of unique diagnostic indications over the years. The percentage increase is statistically significant, with *r*^2^ = 0.38 and *p* < 0.0001. **(C)** Percentage of vasculitic neuropathies compared to the total of unique diagnostic indications year by year. The percentage increase is statistically significant, with *r*^2^ = 0.60 and *p* < 0.0001. **(D)** Percentage of toxic-deficient and metabolic neuropathies compared to the total of univocal diagnostic indications year by year. The percentage decrease is statistically significant, with *r*^2^ = 0.22 and *p* = 0.004. *p < 0.05, **p < 0.01, and ***p < 0.001.

The proportion of hereditary neuropathies significantly decreased over time (*r*^2^ = 0.22, *p* = 0.016, Figure 5A). The percentage of vasculitic and chronic inflammatory neuropathies has instead undergone a statistically significant increase (*r*^2^ = 0.38 and *p* < 0.0001 for chronic inflammatory neuropathies; *r*^2^ = 0.57 and *p* < 0.0001 for vasculitis, Figures 5B,C). On the other hand, toxic-deficient and metabolic neuropathies underwent a percentage reduction (*r*^2^ = 0.22 and *p* = 0.0038, Figure 5D).

Considering 332 patients for whom one or more clinical suspects were available (excluding cases in which clinical suspicion lacked), in 190 (57.2%) clinical suspicion was confirmed by nerve biopsy, while in the remaining 142 cases (42.8%) nerve biopsy modified the clinical diagnosis.

Among 396 patients for whom nerve biopsy did not provide diagnostic indications, in 71.5% (*n* = 283) of cases a precise etiological hypothesis was missing even before histological examination. On the contrary, considering 708 patients with an absent clinical suspicion, in 38.9% of cases (*n* = 275) the histological report was indeterminate, while in 61.1% of cases (*n* = 433) biopsy provided one or more diagnostic indications.

Regarding 52 patients with histological diagnosis of genetic neuropathy in the period from 1991 (year of identification of 17p11.2 chromosome duplication as responsible for CMT1A) (25, 26) to 2017, we assessed how many times diagnosis was molecularly confirmed. We therefore considered 32 patients with histological diagnosis of hereditary neuropathy, excluding 20 patients not molecularly analyzed. In 53.1% (*n* = 17) of cases molecular confirmation of diagnosis was obtained, in 15.6% (*n* = 5) molecular diagnosis was not achieved with a single genetic test, and in the remaining 31.3% (*n* = 10) of patients molecular diagnosis was not achieved after several tests.

**Figure 6:**
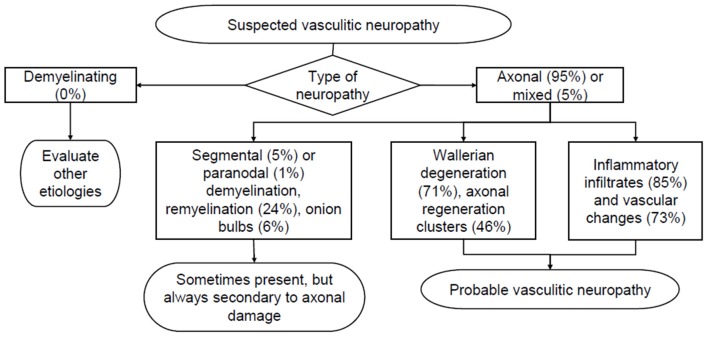
Simplified histological diagnostic pathway in the evaluation of suspected vasculitic neuropathy. The percentages derive from the analysis of 111 biopsies suggestive of vasculitis reported at the Neurological Clinic in the period 1981–2017.

In our series, neuropathies with histological picture suggestive or diagnostic of vasculitis are 111, 95% (*n* = 105) of which axonal and 5% (*n* = 6) with mixed features of demyelination and axonal damage. Active Wallerian degeneration was highlighted in 71% of cases (*n* = 79), demyelination and remyelination were however present in 24% (*n* = 27) of patients, but always secondary to primitive axonal damage. Inflammatory infiltrates were found in 85% (*n* = 81) of cases, with chronic vascular changes predominating in the remaining cases (fibrous obliteration of lumen, calcifications, recanalization and internal elastic lamina fragmentation). In several cases, periavventitial hemosiderin-containing macrophages were found, suggesting previous hemorrhages. Axonal regeneration clusters, suggestive of the end of the acute phase of damage, were highlighted in 46% (*n* = 51) of cases (Figure 6). In 6 cases, basal membrane residues of Schwann cells were arranged to form Bungner bands, expressing initial regeneration. When performed, immunohistochemical analysis (IHC) with stains specific for the antigenic determinants of various inflammatory cells demonstrated infiltrates mainly composed of macrophages (CD68+) and T-lymphocytes (CD45-RO+, CD4+/CD8+). B-lymphocytes (CD20+) were less frequently reported. IHC has replaced direct immunofluorescence (no longer performed since 2010), which was instead used in the past to highlight immunoglobulin, complement and fibrinogen deposits at epineurial vessels wall.

For vasculitis, the ratio between affected females and males was 1.8 (65% females and 35% males). Mean age at biopsy (calculated on 77 patients whose age was known) was 62 years.

Considering 76 patients with known clinical suspicion, in 68% (*n* = 52) vasculitis was already suspected, while in 32% (*n* = 24) nerve biopsy modified the clinical diagnosis.

Since Genoa University is a reference center, many patients come from other centers, so obtaining detailed clinical information is difficult. Among 27 cases of vasculitic neuropathy with clinical records completely available, vasculitis was: HCV-related in 30% (*n* = 8), cryoglobulinemic in 4% (*n* = 1), associated with Churg-Strauss syndrome in 11% (*n* = 3), ANCA-associated with lung and peripheral nervous system (PNS) involvement in 4% (*n* = 1), associated with connectivity's in 11% (*n* = 3), involving skin and PNS in 7% (*n* = 2), and involving kidney and PNS in 4% (*n* = 1) of cases. Finally, vasculitis was limited to PNS in the remaining 30% (*n* = 8) of patients.

In our series, amyloidotic neuropathies are 25 (in one extra case the bioptic material consisted only of vascular structures with amyloid deposits, but the nerve was not assessable), 80% (*n* = 20) of which axonal, with active Wallerian degeneration in 56% (*n* = 14) and regeneration clusters in 36% (*n* = 9) of cases. The remaining 20% (*n* = 5) of biopsies showed coexistence of Wallerian degeneration and segmental or paranodal demyelination. Vascular changes were noted in 40% (*n* = 10) of cases. Constantly found feature was the deposit of amyloid substance in the epineurium, perineurium, and endoneurium. Amyloid fibrils, stained with Congo red, show a typical apple-green birefringence under a polarized light microscope, which allows a certain etiological diagnosis.

## Discussion

Nerve biopsy is often the final step in the diagnostic work-up of neuropathies of unknown origin. Although not generally necessary, biopsy has an essential role in particular situations, identifying specific alterations.

### Epidemiology and Variations Over the Years

Observing the number of annually performed biopsies we found a significant lower number of cases in which biopsy has been prescribed, whereas in the'80 years we had a peak of 60 biopsies, differently in the last years we had a constant number of 20 biopsies per year. This reduction is likely due to the development of alternative diagnostic methods, such as genetic tests and additional laboratory investigations. For example, in dysimmune neuropathies, immunological tests detecting circulating antibodies against myelin (myelin-associated glycoprotein, MAG), axonal (ganglioside), or Ranvier node (155 neurofascin or contactin) components radically changed the diagnostic approach. Furthermore, the percentage of hereditary neuropathies diagnosed by sural nerve biopsy has dropped down, since molecular tests, including next generation sequencing, approaches are now available in most countries (27). Consistently, the mean age of patients undergoing a nerve biopsy significantly decreased. Nowadays, in children and adolescents a sural nerve biopsy is required only in exceptional cases, for instance to orient the genetic/molecular tests and to help establishing the genotype-phenotype correlation (28). Since the mean age of onset of vasculitic neuropathy is 60 years (29), the age increase is also explained by the raised percentage of vasculitic neuropathies in agreement with literature (30). Metabolic and toxic-deficient neuropathies are less represented in recent years and we can speculate that this happened because of the improvement of social and health conditions.

In the first years of the study, in fact, inhalation of glue solvents (N-hexane and methyl-butyl-ketone) and exposure to other industrial toxic substances and heavy metals were certainly more frequent. In 1973 a law was issued (Article 4) for the regulation of the use of chemical reagents in industries and probably after that also the consequences to the exposure have been decreased. However, it must be considered that nerve biopsy is rarely performed in the suspicion of toxic or deficient neuropathies as diagnosis is usually achieved through careful medical history, general and neurological examination, electrophysiology, and laboratory investigations.

Moreover, out data confirm the higher frequency of vasculitic neuropathies in females. This is predictable, since vasculitis is generally immune-mediated and autoimmune diseases have a higher incidence in the female sex (31). In other types of diagnosis, however, the ratio between males and females led to unexpected results. In particular, both vasculitic and amyloidotic neuropathies were diagnosed 12 times more in males than in females. Such a marked gender difference is not described in the literature and probably is an artifact of our pool of data.

#### Vasculitis

Among vasculitic neuropathies, inflammatory infiltrates were found in most cases (85%), but not in all samples, with prevalent chronic vascular changes in the remaining 15%. After the acute phase, inflammatory infiltrates may disappear leaving the pathological hallmarks of fibrous obliteration of lumen, calcifications, recanalization and fragmentation of internal elastic lamina. In several cases, hemosiderin-containing macrophages were found at the periavventitial level, indicating previous bleeding. At the end of the acute phase, axonal regeneration clusters often appear, but they may miss if the loss of fibers was massive. Immunohistochemistry with specific antibodies for the antigenic determinants of inflammatory cells has been frequently used and is still essential for the precise diagnosis of some dysimmune neuropathies. In contrast, direct immunofluorescence, previously used to highlight epineurial deposits of immunoglobulin, complement, and fibrinogen, has not been used in our laboratory since 2010, following the introduction of anti-MAG and anti-gangliosides antibodies plasma dosage.

Non-systemic vasculitic neuropathies represent the 30% of analyzed vasculitic cases. If nerve biopsy is useful, but not always essential in patients with known systemic vasculitis, histological examination is mandatory when PNS-limited vasculitis is suspected.

#### Amyloidotic Neuropathy

Regarding amyloidotic neuropathies, Congo red staining and apple-green birefringence under polarized light microscope allow a certainty diagnosis. It is interesting to note that 20% of biopsies presented mixed features of axonal and myelin damage. The presence of segmental demyelination in amyloidotic neuropathies, better evaluated by teasing, has been previously described by some authors (32–34).

#### Diagnostic Yield of Sural Nerve Biopsy

The diagnostic yield of nerve biopsy is still a topic of discussion. Over the 37 years examined, out of a total of 1,179 sural nerve biopsies, the univocal diagnoses were 52.7%, the cases in which the histology provided multiple diagnostic options 13.7% and indeterminate reports 33.6%. Nerve biopsy was therefore helpful in more than half of patients undergoing this test.

Considering only patients with a specific clinical etiological hypothesis, histological examination confirmed the pre-biopsy suspect in 57.2%, while in the remaining 42.8% of cases nerve biopsy changed clinical diagnosis.

The diagnostic yield showed a progressive improvement, with an increase in the number of univocal diagnoses. This may be explained by the refinement of cases in which a pathological exam is required and by the improving expertise of our center. In our opinion, this supports the view that biopsies should be processed and examined by expert personnel, in order to both reduce artifacts and recognize the pathological hallmarks of different diseases.

Considering indeterminate reports, in 71.5% of cases a precise etiological hypothesis was missing even before biopsy. Even in a retrospective study by Deprez et al., the lowest diagnostic yield was when biopsy was performed in the absence of clinical suspicion (14). Deprez also reported that only 20% of nerve biopsies provided useful information in the absence of a clinical suspicion, highlighting the need for appropriate clinical work-up (20). As a consequence, the importance of carefully selecting patients undergoing nerve biopsy emerges. These results suggest that, when a precise diagnosis not achieved after complete clinical, laboratory and instrumental examinations, nerve biopsy is rarely recommended.

If we considered our cohort of vasculitic neuropathies, in 68% of patients a vasculitis was already suspected before histological examination, while in 32% of cases, the diagnosis was clarified only after the pathological exam nerve biopsy modified clinical diagnosis.

These results about the diagnostic yield confirm what was already known from previous literature (8, 11–14, 35–37).

Our work confirms a rather high diagnostic yield of nerve biopsy, comparable to that obtained by Neundörfer et al. (8) and Gabriel (13). since biopsy was helpful in guiding the diagnosis in more than half of patients.

The diagnostic yield of this exam increases if patients are carefully selected. Comprehensive clinical information are also crucial since in most neuropathies a diagnosis can be obtained by combining the results of biopsy with clinical features. The importance of the choice of a clinically or electrophysiologically affected nerve to be biopsied should be reiterated, since 12% of examined nerves proved to be free of pathological changes. However, in many cases of normal sural nerve, biopsy was actually performed to exclude a peripheral neuropathic process in the context of differential diagnosis from central nervous system or motor neuron diseases.

According to literature, sural nerve biopsy provides the most useful results in interstitial neuropathies, such as vasculitis, granulomatosis, amyloidosis, or atypical forms of CIDP (7, 22). In fact, the greatest diagnostic yield is obtained in asymmetric or multifocal neuropathies, which are the typical features of vasculitis (20).

## Conclusions

This is the largest Italian study evaluating the diagnostic yield of sural nerve biopsy, both for the number of biopsies and for the period considered. Over time there was a progressive refinement of biopsy prescription. Nevertheless, it remains a pivotal exam in interstitial neuropathies, such as amyloidosis and vasculitis, in particular in non-systemic vasculitic neuropathies, allowing a diagnosis and addressing an appropriate treatment.

## Data Availability Statement

All datasets generated for this study are included in the article/Supplementary Material.

## Ethics Statement

All patients signed the informed consent to the processing of the personal data and the use of biopsy samples for research purposes in relation to the laws in force during the period of the intervention, carried out for diagnostic purposes. All tissue samples taken are now stored in the neurologic biobank (Bioneuro) which was established by the Policlinico San Martino IRCCS (Genoa, Italy) with the aim of making tissue samples available to researchers at an international level (http://www.ospedalesanmartino.it/ricerca-scientifica/introduzione-crb/biobanche-e-servizi.html). For this reason, the approval of the Ethical Committee was not required as per the local legislation and national guidelines.

## Author Contributions

VP designed and supervised the study. SM collected and analyzed the data. AG, EB, and PM analyzed the genetic data. MM and EV provided patients data. GM, MG, and AS supervised the study. VP, SM, and GM wrote the article. All authors discussed the results.

### Conflict of Interest

The authors declare that the research was conducted in the absence of any commercial or financial relationships that could be construed as a potential conflict of interest.

## Abbreviations

CMT1A: Charcot Marie Tooth type 1A
CIDP: Chronic Inflammatory Demyelinating Polineuropathy
PNS: Peripheral Nervous System
MAG: Myelin Associated Glycoprotein.
