# Supplementary material for: Diagnostic Value of Sural Nerve Biopsy: Retrospective Analysis of Clinical Cases From 1981 to 2017
Source: Front Neurol. 2019 Nov 22;10:1218. doi: 10.3389/fneur.2019.01218 (PMC6884026; doi:10.3389/fneur.2019.01218)
Supplement: Supplementary file 1 [file Table_1.DOCX]

Supplementary Material

# Supplementary Table
